# Starvation hardiness as preadaptation for life in subterranean habitats

**DOI:** 10.1038/s41598-023-36556-9

**Published:** 2023-06-14

**Authors:** Peter Kozel, Tone Novak, Franc Janžekovič, Saška Lipovšek

**Affiliations:** 1 Department of Biology, Faculty of Natural Sciences and Mathematics, University of Maribor, Koroška Cesta 160, 2000 Maribor, Slovenia; 2 Research Centre of the Slovenian Academy of Science and Arts, Karst Research Institute, Titov Trg 2, 6230 Postojna, Slovenia; 3 Faculty of Medicine, University of Maribor, Taborska Ulica 8, 2000 Maribor, Slovenia; 4 Faculty of Chemistry and Chemical Engineering, University of Maribor, Smetanova Ulica 17, 2000 Maribor, Slovenia

**Keywords:** Biochemistry, Cell biology, Ecology, Zoology, Ecology

## Abstract

Most subterranean habitats, especially caves, are considered extreme environments, mainly because of the limited and erratic food supply and constant darkness. In temperate regions, many climatic conditions, such as temperature and air humidity, are periodically less adverse or even more favourable in caves than the harsh seasonal weather on the surface. Accordingly, many animal species search for hibernacula in caves. These overwintering, non-specialized subterranean species (non-troglobionts) show various modes of dormancy and ongoing development. Since they do not feed, they all undergo periodic starvation, a preadaptation, which might evolve in permanent starvation hardiness, such as found in most specialized subterranean species (troglobionts). To this end, we performed a comparative analysis of energy-supplying compounds in eleven most common terrestrial non-troglobiont species during winter in central European caves. We found highly heterogeneous responses to starvation, which are rather consistent with the degree of energetic adaptation to the habitat than to overwintering mode. The consumption of energy-supplying compounds was strongly higher taxa-dependant; glycogen is the main energy store in gastropods, lipids in insects, and arachnids rely on both reserve compounds. We assume that permanent starvation hardiness in specialized subterranean species might evolved in many different ways as shown in this study.

Animals continuously expend energy to survive, but most do not process food continuously and therefore rely on endogenous physiological fuels^1,2^. Dormant species regularly spend non-feeding periods, i.e., a shorter or longer cessation of feeding, which is typical also for species inhabiting extreme habitats with scarce food supply, such as many caves and other subterranean habitats. In temperate zones, dormant epigean species undergo a starvation period, either in quiescence (i.e., an immediate response to the decline of a limiting environmental factor) or in diapause (i.e., an endogenously programmed fasting that is usually triggered by adverse environmental conditions)^3^. Others, e.g., insects, adopt a mixed strategy alternating torpor and periodic arousal that resemble those of mammalian hibernators^4^. The dormant state is characterized by arrested development, suppressed metabolism, and increased stress responses^3,5–8^. Dormancy forms differ widely in intensity and duration and are difficult to classify; therefore, general agreement on terminology is still lacking^7,9,10^.

One of the most challenging factors for species in many subterranean habitats is the limited and erratic food supply^11–14^, which inevitably leads to shorter or longer periods of starvation. In such conditions, animals are supposed to be very energy cautious^15^, endure to starvation and able to store building and energy-supplying compounds within their bodies to survive^13^. During extended starvation, stored lipids, mostly triacylglycerols, and glycogen, are essential energy-supplying compounds in animals^3,16–18^. Reserve proteins, mostly in the form of protein granules, supply amino acids; in many invertebrates, spherites store and supply essential minerals; and other metabolites, such as circulating sugars and hexameric proteins, provide additional compounds essential for survival during starvation periods^3,16–19^. Most lipids are stored in specialized cells, such as R cells in crustaceans, and adipocytes in the midgut, fat body, muscles, Malpighian tubules, oleospheres and other organs in other arthropods^12,16,20,21^. Glycogen is stored in many cell types and organs, such as the midgut, fat body, muscles and hepatopancreas^16,22,23^. In cells, triacylglycerols are stored in the form of small to huge droplets of up to 0.5 mm in diameter^12^. Glycogen is present in the form of individual granules, glycogen rosettes and glycogen vesicles^24,25^.

Beside specific morphological adaptations to the subterranean habitat, such as eyelessness, appendage modification and depigmentation^26^, adaptations in specialized subterranean species (troglobionts) include also improved ability in other senses, longevity and specific physiological, ethological, and other adaptations with respect to traits in the epigean congeners^14,27–29^. Non-troglobionts (i.e., trogloxenes and troglophiles in an early, Schiner-Racoviţă ecological classification of subterranean organisms^11,14,24,30^) are expected to show either some exaptations (i.e., traits suitable for a function other than that for which it was developed through natural selection^31^) or preadaptations (i.e., features that fortuitously serve a new function^32^) for life in the subterranean habitat. Therefore, this is a promising group to study adaptation processes to the subterranean habitat. Recently, there have been some attempts to provide reviews on functional traits of the subterranean fauna, e.g., in fishes^33^, amphipods^34–36^ and spiders^37^. Moretti et al.^38^ provided a manual for measuring functional traits in epigean terrestrial invertebrates that may serve well to fill knowledge gaps when considering invertebrates in subterranean habitats.

Some aquatic subterranean species, e.g., some amphipods, have greater reserves of energy-supplying compounds and sustain lower metabolic rates than related epigean taxa^39–42^. They all store energy-supplying compounds, while low metabolic rate is not a universal property of specialised subterranean animals, and the degree of metabolic adaptation is not always in agreement with the degree of morphological adaptation, e.g., in some amphipods and aquatic isopods^42,43^. However, reports on the energy reserves in terrestrial subterranean invertebrates are limited to non-troglobionts. Only a few of them have been studied for their energy metabolism; lipid-dependant metabolism predominates in most of them^20,21,24,25,44–50^. Lipid dynamics can be one of the clues for estimating the physiological state and overall condition of an organism during starvation. Such data provide insights into the balance between catabolism and synthesis during periods of fasting^41^. It is estimated that in central Europe, there are at least fifty terrestrial invertebrate species frequenting caves with no apparent, or with weakly expressed specific adaptation to the habitat^51^. Among these, eleven species that regularly spend winter in central European caves, make up the bulk of macrofauna (body size > 2 mm) with respect to abundance, body mass and energy stores^21,52^.

According to hitherto studies, beside quiescence and diapause, some non-troglobiont terrestrial invertebrate species conduct a nonfeeding, prevalently resting overwintering accompanied by growth, moulting and gonad ripening in caves^45,53–56^. Moreover, some other non-troglobionts that otherwise feed in caves throughout the year when food is available, undergo quiescence- and dormancy-resembling starvation and resting because of the rare opportunities to feed^45^. Thus, responses of organisms to low food supply are heterogeneous, as reported also for other traits^14^. Accordingly, in this contribution, we use the term overwintering in the broadest meaning referring to spending the wintertime.

Starvation hardiness can be considered as one of clue traits to living in energy-poor subterranean habitats. For epigean species, prolonged starvation during periodical dormancy in subterranean habitats provides a potential opportunity to evolve steady hardiness to a limited and erratic food supply. In this study, we provide an overview of the dynamics of energy-supplying compounds, and a comparative analysis of these compounds in eleven most common terrestrial non-troglobiont species overwintering in central European caves. To this end, we compiled hitherto published and new evidence on energy-supplying compounds in these species. Our goal was to find evidence for the evolution of steady starvation hardiness. We hypothesized that these species show heterogeneous response to starvation in three main directions: (a) low consumption of energy-supplying compounds in species maintaining basal metabolism along with rigorous ceasing any activity during overwintering, (b) moderate consumption in year-round active, opportunistic feeding species, and (c) high consumption of the compounds due to ongoing maturation processes resulting in immediate reproduction ability in spring.

## Materials and methods

### Species studied

Specimens of eleven non-troglobiont species of both sexes (Table 1), except the hermaphrodites and juveniles of unidentified sex, for biometrical, biochemical, and cytological consideration were collected between 2003 and 2011 from seven caves in Slovenia and one cave in north-eastern Italy^52^. Among these, *M. menardi* and *L. schreibersii* are troglophiles, and all the others are trogloxenes according to Schiner-Racoviţă classification^57^. *Meta menardi* and *L. schreibersii* (in favourable conditions) feed throughout the year, while all the other species cease completely feeding during overwintering. Following published guidelines^7,10,49^ and own observations referring to selected ecological, ethological and developmental traits, we allocated species into three functional guilds according to the movement (torpor, movement triggered by disturbance, autogenous movement), state of ontogenesis (arrested, continued) and feeding (fasting, opportunistic feeding) during wintertime in caves. These are the following guilds: a) Guild 1: species in diapause (*C.* (*O.*) *lefeburiana*^48^, *C. illyrica*, *G. annulatus*^47,49^, *G. titanus*^47,49^, *S. libatrix*^20^, *T. neglectus*^55,56^); b) Guild 2: species with ongoing ontogenesis under fasting conditions (*A. aurantiacus*^44,54^, *T. dubitata*^53^, *T. cavicola*^55,56^); and c) Guild 3: troglophiles feeding at occasional opportunities (*M. menardi*^21,27,50,51,58,59^, *L. schreibersii*).

**Table 1:** Species examined for fresh and dry mass, and lipid, glycogen and water content during overwintering with caves and winter of collection.

Higher taxa	Family	Species	Caves*; winter
Mollusca, Gastropoda	Helicidae	*Campylea* (*Oricampylea*) *lefeburiana* (A. Férussac, 1821)	1; 2005–2006
		*Campylaea illyrica* (Stabile, 1864)	7, 8; 2007–2008
Arachnida, Opiliones	Phalangiidae	*Amilenus aurantiacus* (Simon, 1881)	6; 2006–2007
		*Gyas annulatus* (Olivier, 1791)	2, 4; 2003–2004
		*Gyas titanus* Simon, 1879	2, 4; 2003–2004
Arachnida, Araneae	Tetragnathidae	*Meta menardi* (Latreille, 1804)	6, 7; 2005–2006
Insecta, Coleoptera	Carabidae	*Laemostenus schreibersii* (Küster, 1846)	5; 2010–2011
Insecta, Lepidoptera	Geometridae	*Triphosa dubitata* (Linnaeus, 1758)	6, 7; 2005–2006
	Erebidae	*Scoliopteryx libatrix* (Linnaeus, 1758)	6, 7; 2005–2006
Insecta, Orthoptera	Rhaphidophoridae	*Troglophilus cavicola* (Kollar, 1833)	3; 2004–2005
		*Troglophilus neglectus* Krauss, 1879	3; 2004–2005

*1 Abisso Bonetti (45.834167 N, 13.581389 E), 2 Babja luknja pri Goričanah (46.13425° N, 14.39185° E), 3 Špegličeva jama (46.2993° N, 15.19446° E), 4 Rački pekel (46.3904° N, 14.71616° E), 5 Huda luknja (46.41443° N, 15.17433° E), 6 Špehovka (46.41554° N, 15.17276° E), 7 Karbelova jama (46.62852° N, 15.06361° E), 8 Jama pod Herkovimi pečmi (46.62591° N, 15.26323° E).

### Laboratory analyses

Specimens were measured for their size, fresh and dry mass, and lipid, glycogen, and water content at the beginning (November), in the middle (January) and at the end of overwintering (March) after sacrifice by exposure to –20 °C for two hours. Numbers of individuals studied are given in Table 2. The dry mass was determined after 15 days of vacuum desiccation under P_2_O_5_. Lipids were extracted from completely macerated, dry individual samples via diethyl ether extraction^60^ by addition of 2 mL diethyl ether, stirring for 5 min, decanting ether and being weighed after 24 h desiccation of air-dried sample under P_2_O_5_. Larger samples with more fats were eluated twice. Glycogen was quantified in the same samples after lipid extraction with the anthrone reaction^61^, by putting a sample in 2 mL of KOH (300 g L^–1^), and boiled for 20 min while shaking. After cooling, the undissolved particles were sedimented by centrifugation (1000 × g, 5 min). The supernatant was decanted, cooled in an ice bath, and mixed with 0.2 mL of saturated (NH_4_)_2_SO_4_. Glycogen was precipitated by adding 5 mL of 96% ethanol and cooled in an ice bath for 5 min. After centrifugation (1000 × g, 5 min), the supernatant was discharged, and the sedimented glycogen dried at 105 °C and weighed. Then it was dissolved in 4 mL of water by warming to boiling point. 2 mL of the anthrone reagent (2 g L^–1^ in conc. H_2_SO_4_) was added to 0.5 mL of the glycogen solution and warmed in a boiling water bath for 10 min. After cooling, the absorption was recorded at 620 nm against a reagent blank. The glucose solution (0.1 g L^–1^) was used as the reference, and a correction factor of 0.9 was used in calculations of the glycogen amounts.

**Table 2:** Number of specimens (N), mean ± StD, median and IQR of fresh and dry mass, and lipid, glycogen and water content in the three overwintering periods for the eleven examined species. Variables with non-normally distributed data are in bold.

Species	Period	N	Fresh mass/ individual [mg]	Dry mass / individual [mg]	Lipids/ individual [mg]	Lipids/ dry mass [mg‧g^-1^]	Glycogen/ individual [mg]	Glycogen/ dry mass [mg‧g^-1^]	Water [mg‧g^-1^ fresh mass]
*C. (O.) lefeburiana*	Beginning	5	2202.40 ± 253.73 2155.70, 231.30	328.66 ± 35.06 324.40, 43.00	10.42 ± 5.04 10.10, 7.00	31.65 ± 15.33 31.13, 17.56	5.94 ± 1.00 5.40, 0.76	18.27 ± 3.71 17.13, 2.75	849.71 ± 19.02 849.52, 9.87
	Middle	10	1472.95 ± 352.61 1482.30, 171.45	249.62 ± 62.18 241.30, 52.02	6.76 ± 2.52 6.45, 3.22	27.60 ± 8.95 26.08, 14.15	4.47 ± 2.23 4.40, 1.40	17.47 ± 6.70 15.66, 7.29	843.86 ± 9.65 843.09, 9.38
	End	6	1582.60 ± 376.43 1451.75, 425.35	218.20 ± 39.96 216.90, 44.77	6.63 ± 1.98 6.85, 2.70	30.96 ± 10.39 28.43, 16.18	9.63 ± 5.57 9.28, 7.55	43.15 ± 19.98 49.23, 27.50	872.28 ± 8.12 872.83, 5.38
*C. illyrica*	Beginning	5	1944.28 ± 43.64 1928.60, 20.30	**237.60 ± 31.47** **221.10, 18.10**	5.28 ± 0.90 5.60, 1.30	22.33 ± 3.67 20.09, 6.11	**9.01 ± 5.93** **7.35, 1.98**	36.29 ± 17.81 33.63, 11.05	**878.01 ± 13.34** **885.36, 9.04**
	Middle	10	1768.68 ± 440.09 1737.30, 533.78	293.03 ± 57.42 288.80, 31.35	**9.02 ± 4.33** **7.45, 4.97**	29.90 ± 9.87 27.10, 7.90	7.96 ± 4.07 6.42, 6.92	26.77 ± 12.36 25.45, 13.80	832.73 ± 17.29 829.01, 24.61
	End	5	1672.50 ± 77.19 1684.30, 10.20	298.10 ± 22.21 287.10, 32.70	5.66 ± 1.07 5.50, 1.30	19.15 ± 4.19 19.91, 2.43	6.61 ± 2.42 5.92, 0.68	**22.40 ± 9.03** **18.59, 4.36**	**702.25 ± 9.32** **702.25, 11.79**
*A. aurantiacus*	Beginning	10	17.51 ± 3.54 16.90, 4.27	7.72 ± 1.63 7.25, 1.95	1.84 ± 0.74 1.75, 1.07	230.67 ± 48.01 225.81, 87.67	0.17 ± 0.05 0.17, 0.04	**22.25 ± 8.18** **20.79, 3.30**	559.65 ± 9.91 556.92, 10.05
	Middle	20	21.19 ± 2.30 20.65, 2.95	5.98 ± 0.97 6.00, 1.33	1.24 ± 0.59 1.15, 0.70	213.00 ± 106.49 207.46, 120.45	0.05 ± 0.03 0.05, 0.04	9.14 ± 5.03 9.37, 7.24	716.05 ± 48.04 706.51, 70.54
	End	10	19.17 ± 3.56 18.25, 5.80	5.29 ± 1.00 5.10, 0.97	0.59 ± 0.34 0.55, 0.28	106.86 ± 47.10 100.74, 61.08	**0.08 ± 0.01** **0.07, 0.02**	15.90 ± 5.06 14.52, 7.45	723.41 ± 18.53 726.64, 23.52
*G. annulatus*	Beginning	10	29.89 ± 5.29 29.30, 8.40	7.91 ± 2.32 7.20, 2.15	0.95 ± 0.59 0.85, 0.68	117.93 ± 58.11 108.79, 69.82	0.06 ± 0.01 0.06, 0.01	8.51 ± 1.86 8.57, 2.28	737.38 ± 41.76 752.41, 63.30
	Middle	20	30.32 ± 6.18 30.10, 9.40	9.21 ± 2.35 9.25, 3.33	0.81 ± 0.30 0.82, 0.41	109.90 ± 37.91 115.58, 42.31	0.04 ± 0.01 0.05, 0.01	5.65 ± 1.35 5.65, 1.38	747.15 ± 34.47 746.32, 45.98
	End	10	30.72 ± 4.24 31.93, 6.82	**7.42 ± 1.02** **7.10, 0.24**	0.76 ± 0.18 0.83, 0.23	101.86 ± 17.70 100.68, 24.79	0.02 ± 0.01 0.02, 0.01	2.78 ± 0.84 2.74, 0.88	756.92 ± 27.17 754.85, 25.83
*G. titanus*	Beginning	10	36.73 ± 4.27 35.00, 4.77	12.02 ± 1.84 11.40, 2.40	2.51 ± 0.78 2.55, 0.90	207.96 ± 51.90 223.64, 73.93	0.03 ± 0.01 0.03, 0.01	2.79 ± 0.75 3.01, 1.01	673.89 ± 13.46 672.41, 22.67
	Middle	10	36.98 ± 4.77 36.91, 4.18	11.66 ± 0.70 11.54, 0.58	2.33 ± 0.90 2.53, 0.69	203.16 ± 83.42 215.43, 71.84	0.03 ± 0.01 0.03, 0.01	2.32 ± 0.44 2.13, 0.66	696.30 ± 33.12 696.66, 35.69
	End	10	37.53 ± 9.31 37.83, 14.28	10.50 ± 2.66 10.00, 3.30	1.56 ± 0.56 1.80, 0.78	147.74 ± 43.25 155.65, 65.99	0.01 ± 0.00 0.01, 0.01	1.32 ± 0.54 1.26, 0.75	720.45 ± 21.18 718.80, 34.46
*M. menardi*	Beginning	10	248.18 ± 80.24 226.85, 142.20	109.70 ± 52.84 106.90, 68.30	14.57 ± 3.43 16.15, 5.37	148.02 ± 39.35 152.29, 45.97	0.21 ± 0.13 0.16, 0.22	1.77 ± 0.49 1.67, 0.55	573.16 ± 86.50 595.95, 130.47
	Middle	21	278.49 ± 94.53 242.60, 66.30	105.81 ± 44.60 108.40, 54.20	**8.01 ± 8.20** **4.90, 4.80**	**70.76 ± 48.28** **61.12, 47.16**	0.19 ± 0.10 0.16, 0.13	**1.94 ± 1.07** **1.77, 0.99**	624.43 ± 84.05 631.04, 106.44
	End	12	290.58 ± 89.52 322.10, 135.57	109.28 ± 46.57 107.40, 58.58	11.16 ± 7.08 12.40, 13.80	104.46 ± 67.51 90.99, 98.04	0.23 ± 0.14 0.25, 0.19	1.99 ± 0.97 1.87, 1.43	633.12 ± 70.19 645.98, 72.28
*L. schreibersii*	Beginning	9	56.44 ± 11.42 55.17, 11.60	22.11 ± 9.19 18.80, 9.63	7.67 ± 6.17 5.60, 6.67	310.68 ± 154.07 339.39, 271.28	0.03 ± 0.01 0.04, 0.01	1.69 ± 0.92 1.64, 1.75	591.92 ± 96.27 605.81, 156.73
	Middle	19	58.67 ± 16.09 57.80, 19.30	23.57 ± 10.09 23.20, 9.40	5.59 ± 4.13 4.80, 6.15	220.43 ± 108.87 243.65, 181.76	**0.10 ± 0.07** **0.09, 0.08**	4.23 ± 2.09 4.31, 1.72	611.42 ± 87.58 586.41, 93.14
	End	10	60.63 ± 19.39 56.40, 25.48	21.34 ± 5.62 21.35, 8.80	**6.79 ± 8.00** **3.55, 3.08**	**288.61 ± 287.81** **168.91, 189.45**	**0.15 ± 0.08** **0.19, 0.12**	7.83 ± 4.89 8.32, 7.34	642.25 ± 38.97 640.92, 32.01
*T. dubitata*	Beginning	10	58.80 ± 7.57 58.70, 10.33	26.42 ± 3.72 26.70, 5.58	3.81 ± 1.02 3.90, 1.63	145.34 ± 39.45 148.22, 62.59	**0.22 ± 0.16** **0.16, 0.13**	**8.78 ± 7.38** **5.71, 4.03**	551.19 ± 11.58 550.19, 10.33
	Middle	21	55.19 ± 12.92 55.60, 12.60	24.34 ± 8.62 24.90, 7.40	**3.01 ± 1.69** **2.80, 2.10**	**151.93 ± 139.34** **124.46, 59.84**	**0.15 ± 0.13** **0.10, 0.12**	**6.77 ± 4.92** **4.21, 5.54**	**562.68 ± 94.06** **564.36, 46.40**
	End	11	50.61 ± 8.40 47.90, 12.75	22.25 ± 4.45 21.40, 7.00	**3.96 ± 3.84** **2.20, 2.10**	**178.62 ± 159.12** **100.00, 104.61**	0.05 ± 0.02 0.06, 0.04	2.38 ± 1.04 1.97, 1.66	561.79 ± 30.66 555.29, 33.37
*S. libatrix*	Beginning	10	261.13 ± 33.56 270.40, 56.28	144.13 ± 18.17 147.55, 14.85	45.37 ± 10.16 45.80, 11.45	**314.06 ± 54.99** **306.21, 57.26**	**2.13 ± 2.28** **1.49, 1.07**	**14.29 ± 14.12** **10.92, 8.06**	445.37 ± 60.16 447.44, 52.64
	Middle	28	218.61 ± 45.72 220.25, 59.30	122.82 ± 39.25 108.00, 30.07	20.98 ± 9.71 19.30, 12.75	163.22 ± 61.22 185.47, 70.17	**1.41 ± 1.36** **0.58, 1.31**	**10.27 ± 7.46** **4.85, 8.86**	493.56 ± 106.23 515.65, 136.66
	End	10	228.82 ± 26.66 238.25, 41.58	121.08 ± 26.35 126.90, 31.57	26.11 ± 14.85 26.35, 9.13	204.90 ± 96.70 192.00, 77.68	0.58 ± 0.30 0.53, 0.48	4.99 ± 2.56 5.59, 3.76	474.86 ± 78.89 489.91, 58.62
*T. cavicola*	Beginning	11	506.61 ± 58.47 509.20, 36.70	168.64 ± 23.91169.00, 19.95	31.55 ± 8.89 30.60, 11.85	185.47 ± 37.34 184.93, 49.12	0.99 ± 0.48 0.81, 0.80	5.80 ± 2.62 4.87, 3.75	667.42 ± 25.90 673.43, 29.48
	Middle	10	539.89 ± 30.64 543.55, 19.65	167.94 ± 10.12 164.75, 9.58	32.74 ± 7.70 32.15, 9.00	193.82 ± 36.40 200.40, 51.47	0.92 ± 0.49 0.88, 0.71	5.54 ± 3.09 5.16, 4.47	688.54 ± 17.47 693.64, 18.39
	End	10	603.90 ± 47.88 611.90, 55.30	141.98 ± 11.36 141.55, 16.43	19.48 ± 9.17 19.85, 13.35	135.20 ± 58.50 143.37, 85.49	1.04 ± 0.39 1.01, 0.62	7.22 ± 2.30 7.43, 3.75	764.66 ± 10.72 762.15, 17.37
*T. neglectus*	Beginning	9	585.02 ± 134.68 557.30, 129.70	175.60 ± 43.23 176.50, 20.40	49.39 ± 16.77 54.50, 21.80	275.18 ± 54.60 282.67, 55.56	0.82 ± 0.35 0.75, 0.54	4.76 ± 1.68 4.90, 3.02	698.79 ± 47.08 702.42, 27.87
	Middle	7	535.76 ± 119.30 527.30, 102.90	165.96 ± 47.32 154.10, 62.45	34.70 ± 15.70 27.20, 23.05	203.81 ± 49.17 232.95, 74.47	0.64 ± 0.48 0.38, 0.64	3.93 ± 2.47 3.02, 3.73	691.05 ± 38.31 691.73, 50.68
	End	8	373.74 ± 178.93 339.20, 96.63	96.16 ± 42.72 90.90, 34.35	13.59 ± 8.02 12.90, 8.05	145.89 ± 56.09 135.65, 71.04	0.62 ± 0.51 0.45, 0.42	**5.89 ± 3.32** **4.54, 2.39**	736.79 ± 25.03 731.74, 23.83

Additionally, three to five specimens of each species in each period were analysed histologically and cytologically in control (details in^25,47,49^). For transmission electron microscopy (TEM), small pieces of the tissue were fixed, post-fixed and dehydrated using standard procedure before being embedded in TAAB epoxy resin. Unfortunately, TEM analysis of gastropods was not possible because during sectioning procedure on microtome, abundant, hard spherites were torn in large numbers out of slices, causing extensive damage in other sample portions; such slices were useless for the TEM analysis. Ultra-thin sections (75 nm) were analysed by a Zeiss EM 902 transmission electron microscope (details in^45^).

### Statistical analysis

Due to relatively small sample sizes for some species, data for males and females were pooled for statistical analyses. The data distribution for fresh and dry mass, amount of energy-supplying compounds, and water content for all species for the three overwintering periods was first checked for normality using the Shapiro–Wilk test. As some data were non-normally distributed, we summarized the data by means, standard deviations (StD), medians and interquartile ranges (IQR). We examined differences in energy-supplying compounds and water content among species, among higher taxa, and among guilds. For this purpose, we used the one-way ANOVA or Welch’s ANOVA (for data with unequal variance) for normally distributed data, and the Kruskal–Wallis test for non-normally distributed data. Where statistically significant differences were found, Tukey HSD post hoc test (for normally distributed data) and the Bonferroni-adjusted Mann–Whitney U test (for non-normally distributed data) were applied. To visualize lipid, glycogen and water content of individual species, higher taxa and guilds, we provided PCA biplots with polygons obtained by the Convex-Hull method. For this purpose, data for individual species, higher taxa and guilds across the three overwintering periods were merged. All tests and PCA biplots were performed in PAST 4.03 program^62^, while dot plots were created with the ggplot2 R package^63^ using the ggplot command.

To summarize energy demands during overwintering in species under study, we calculated average per-day consumption and accumulation of the main energy-supplying reserve compounds, respectively. In the calculations, the value 39 kJ g^–1^ was used for reserve lipids and 17 kJ g^–1^ for glycogen^61^.

## Results

### Dynamics of energy-supplying compounds and water content in the studied species during overwintering

Descriptive statistics for fresh and dry mass, and lipid, glycogen, and water content during overwintering for the eleven species is presented in Table 2. In the following text, only statistically significant differences in lipid, glycogen and water content, obtained by various statistical tests, are reported; the results of the post-hoc tests are presented in Supplementary Table S1.

**Figure 1:**
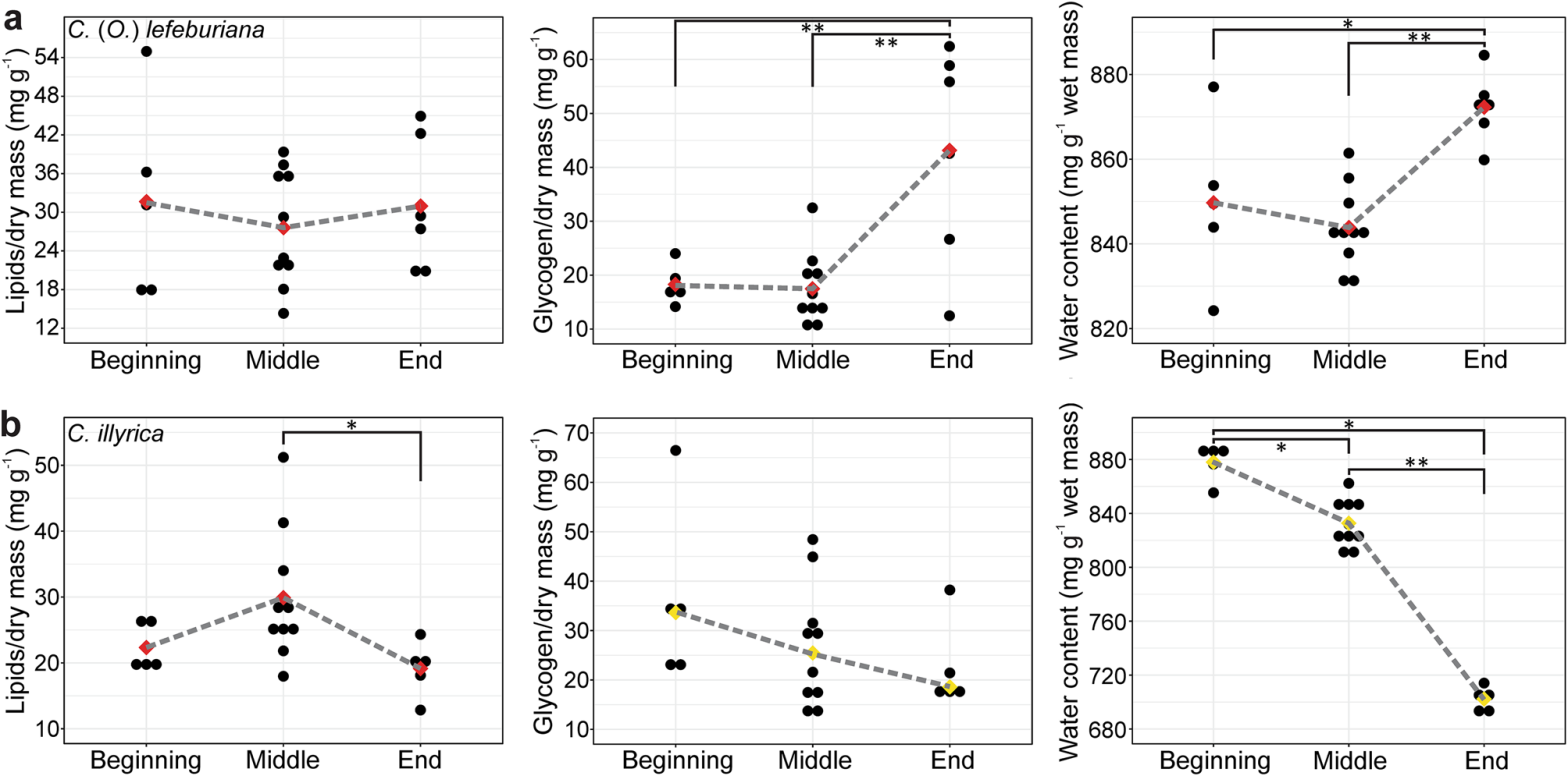
Lipid, glycogen, and water content in *C.* (*O.*) *lefeburiana* (**a**) and *C. illyrica* (**b**) in the three overwintering periods. The red and yellow diamonds represent the mean and the median, respectively. For detailed statistical results see Supplementary Table S1. *p < *0.05*, **p < *0.01*, ***p < *0.001*.

In *C.* (O*.*) *lefeburiana*, we observed statistically significant differences in glycogen (*F*_*9.76*_ = *4.39*, *p* = *0.044*) and water content (*F*_*2*_ = *10.72*, *p* = *0.001*) in three overwintering periods. Both were higher at the end than at the beginning and in the middle of overwintering (Fig. 1a).

In *C. illyrica*, significant differences were found in lipid (*F*_*2*_ = *3.78*, *p* = *0.044*) and water (*χ*^2^ = *15.65*, *p* < *0.001)* content during overwintering. Lipid content was significantly higher in the middle compared to the end of overwintering. Water content was significantly lower in the middle and at the end, compared to the beginning of overwintering, and also lower at the end, compared to the middle of overwintering (Fig. 1b).

**Figure 2:**
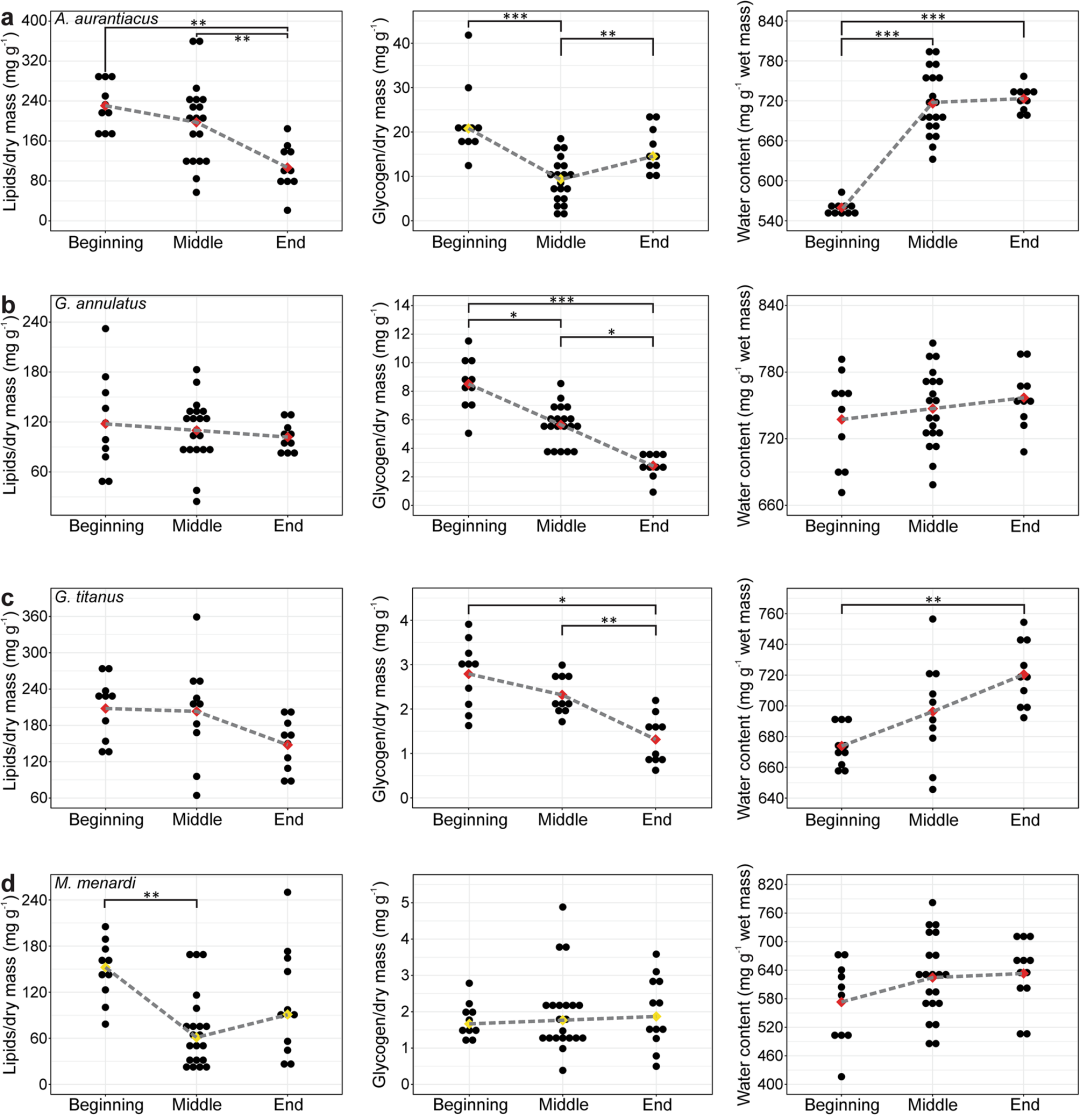
Lipid, glycogen, and water content in *A. aurantiacus* (**a**), *G. annulatus* (**b**), *G. titanus* (**c**) and *M. menardi* (**d**) in the three overwintering periods. The red and yellow diamonds represent the mean and the median, respectively. For detailed statistical results, see Supplementary Table S1. *p < *0.05*, **p < *0.01*, ***p < *0.001*.

In *A. aurantiacus*, lipid content decreased gradually and differed significantly among the periods (*F*_*2*_ = *6.95*, *p* = *0.003*). At the beginning, it was higher than in the middle and at the end of overwintering. Significant differences were also found in glycogen content by period (*χ*^2^ = *20.56*, *p* < *0.001*). More glycogen was present at the beginning and end than in the middle of overwintering. Water content increased over the course of overwintering and differed significantly among the periods (*F*_*21.5*_ = *351.2*, *p* < *0.001*); amounts were significantly higher in the middle and at the end as compared to the beginning of overwintering (Fig. 2a).

Glycogen content in *G. annulatus* gradually diminished during overwintering. We found significant differences among the periods (*F*_*2*_ = *41.99*, *p* < *0.001*). A post-hoc test revealed lower glycogen content in the middle and at the end than at the beginning of overwintering, and lower at the end than in the middle of overwintering (Fig. 2b).

In *G. titanus*, we found significant differences in glycogen (*F*_*2*_ = *16.24*, *p* < *0.001*) and water (*F*_*2*_ = *9.42*, *p* = *0.001*) content during overwintering. Glycogen content was significantly lower at the end compared with the beginning and the middle of overwintering, while water content was significantly higher at the end than at the beginning of overwintering (Fig. 2c).

In *M. menardi*, we found significant differences in lipid content among the periods (*χ*^2^ = *11.91*, *p* = *0.003*). Lipid content was lower in the middle than at the beginning of overwintering (Fig. 2d).

**Figure 3:**
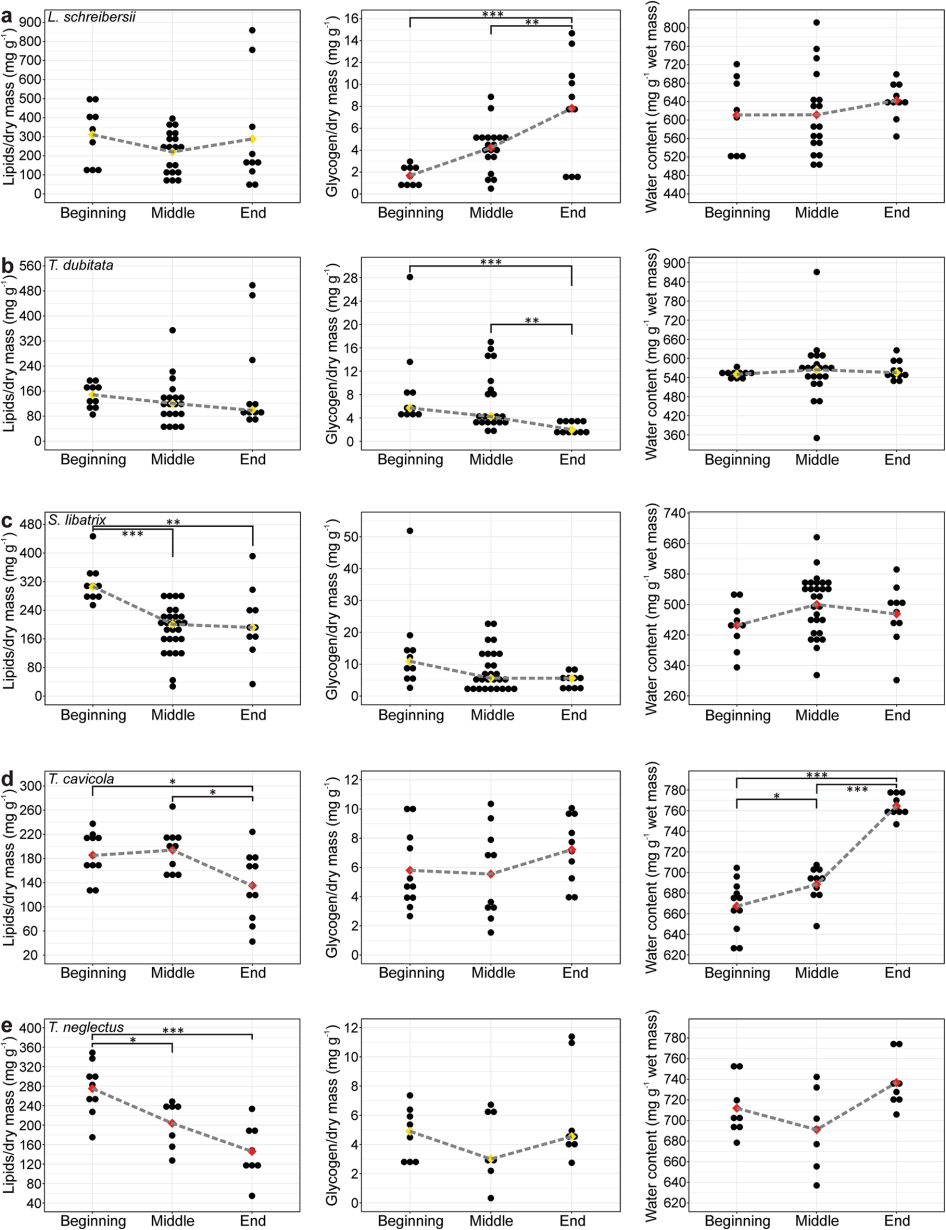
Lipid, glycogen, and water content in *L. schreibersii* (**a**), *T. dubitata* (**b**), *S. libatrix* (**c**), *T. cavicola* (**d**) and *T. neglectus* (**e**) in the three overwintering periods. The red and yellow diamonds represent the mean and the median, respectively. For detailed statistical results, see Supplementary Table S1. *p < *0.05*, **p < *0.01*, ***p < *0.001*.

In *L. schreibersii*, we observed significant differences in glycogen content during overwintering (*F*_*18.3*_ = *15.40*, *p* < *0.001*). Glycogen content was higher at the end than at the beginning and in the middle of overwintering (Fig. 3a).

In *T. dubitata*, there were significant differences in glycogen content among overwintering periods (*χ*^2^ = *18.49*, *p* < *0.001*). Glycogen content was higher at the beginning and in the middle than at the end of overwintering (Fig. 3b).

In *S. libatrix*, we found statistically significant differences in lipid content (*χ*^2^ = *18.03*, *p* < *0.001*) among the time periods. Lipid content was significantly higher at the beginning than in the middle and at the end of overwintering (Fig. 3c).

In *T. cavicola*, there were statistically significant differences in lipid content among the periods (*F*_*2*_ = *5.02*, *p* = *0.014*). Lipid content was significantly higher at the beginning and in the middle than at the end of overwintering. We also found significant differences in water content among the periods (*F*_*2*_ = *71.84*, *p* < *0.001*). The water content was higher at the end than at the beginning and in the middle of overwintering, and higher in the middle than at the beginning of overwintering (Fig. 3d).

In *T. neglectus*, lipid content differed significantly between periods (*F*_*2*_ = *12.41*, *p* < *0.001*). Lipid content was higher at the beginning than in the middle and at the end of overwintering (Fig. 3e).

**Figure 4:**
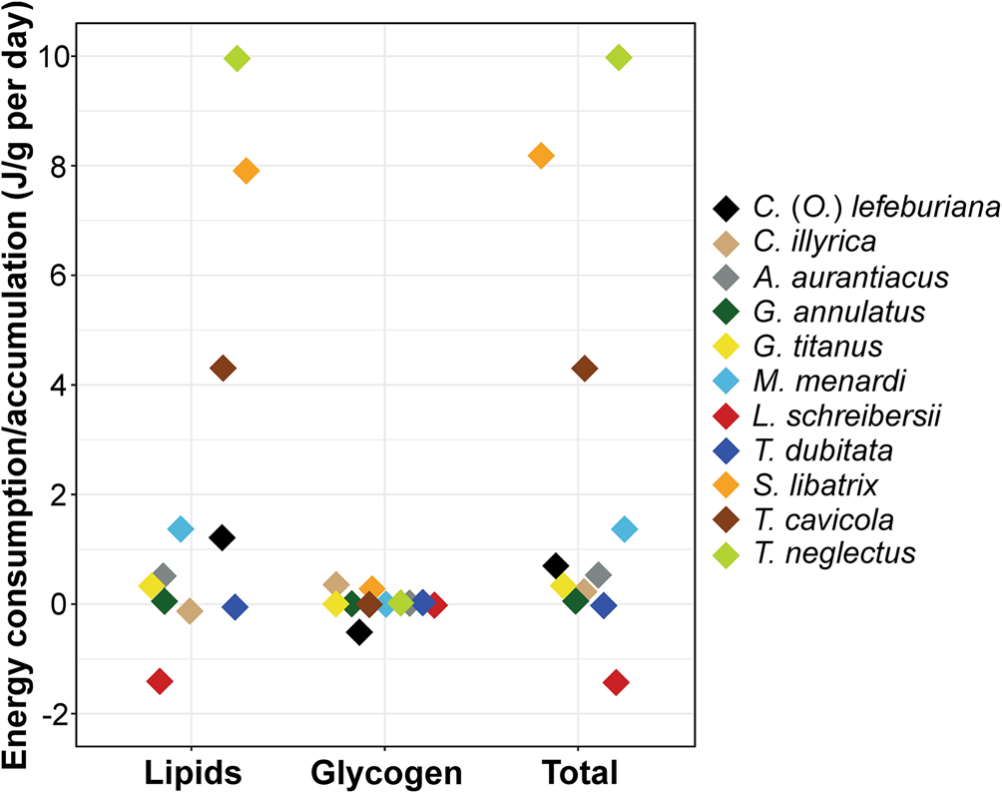
Average consumption (positive values) and accumulation (negative values) of energy-supplying compounds per day in the eleven species during overwintering.

Figure 4 summarizes energy demands in the eleven species during overwintering. Three species: *T. neglectus*, *S. libatrix* and *T. cavicola* showed moderate to high consumption (> 4 J/g per day); six species: *M. menardi*, *C.* (*O.*) *lefeburiana*, *A. aurantiacus*, *G. titanus*, *C. illyrica*, *G. annulatus* and *T. dubitata* with negligible to low consumption (< 2 J/g per day); while *L. schreibersii*, accumulated (> 1 J/g per day) energy-supplying compounds in winter.

### Dynamics of energy-supplying compounds and water content in higher taxa and guilds during overwintering

**Figure 5:**
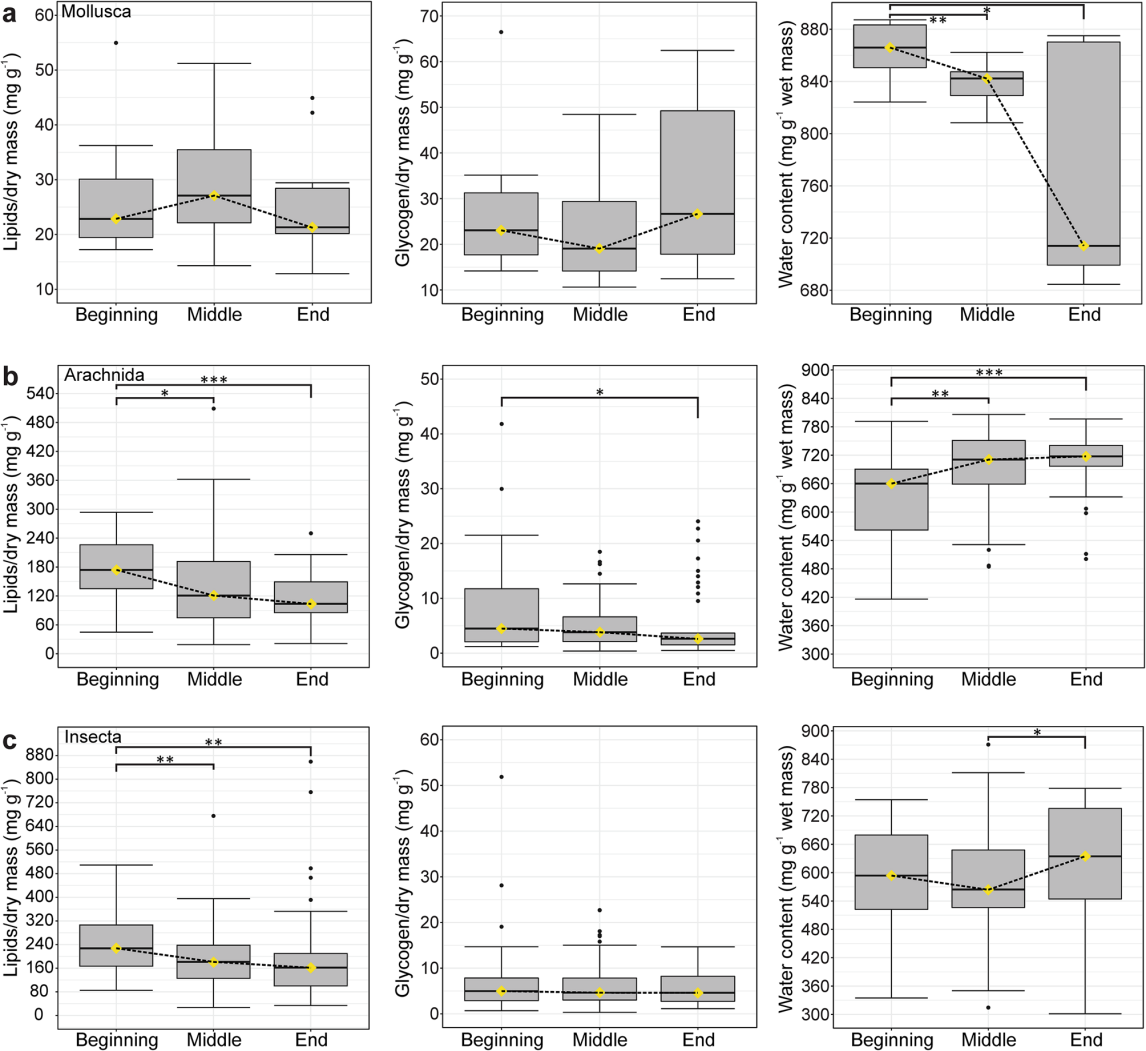
Summary box-whisker plots of lipid, glycogen, and water content in Mollusca (**a**), Arachnida (**b**) and Insecta (**c**) in the three overwintering periods. The yellow diamonds represent the median. For additional statistical findings, see Supplementary Table S2. * *p* < *0.05*, ** *p* < *0.01*, *** *p* < *0.001*.

**Figure 6:**
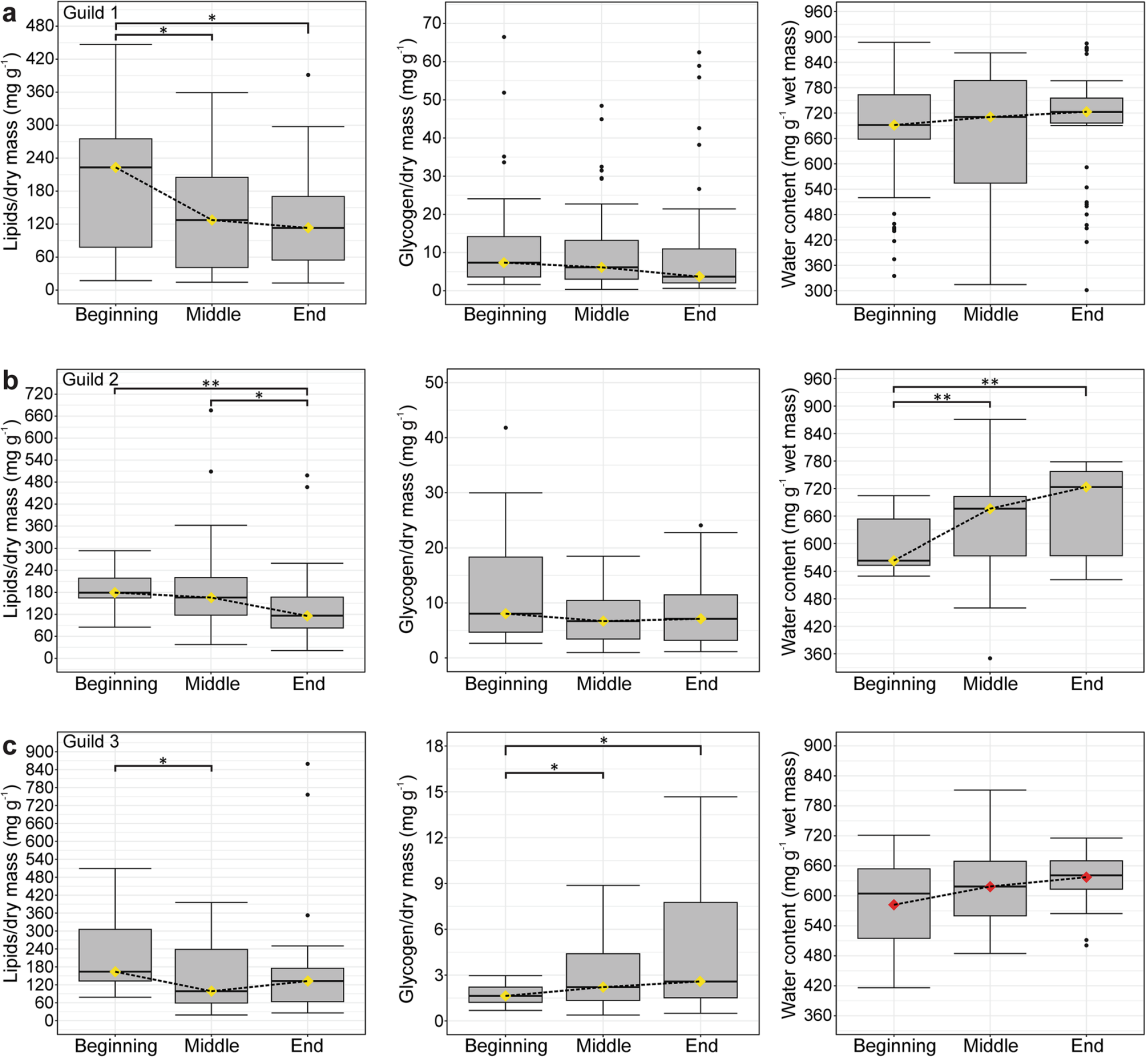
Summary box-whisker plots of lipid, glycogen, and water content in Guild 1 (*C.* (*O.*) *lefeburiana*, *C. illyrica*, *G. annulatus*, *G. titanus*, *S. libatrix*, *T. neglectus*) (**a**), Guild 2 (*A. aurantiacus*, *T. dubitata*, *T. cavicola*) (**b**) and Guild 3 (*M. menardi*, *L. schreibersii*) (**c**) in the three overwintering periods. The yellow and red diamonds represent the mean and the median, respectively. For additional statistical findings, see Supplementary Table S2. * *p* < *0.05*, ** *p* < *0.01*, *** *p* < *0.001*.

Figures 5 and 6 show the general trends in lipid, glycogen, and water content during overwintering for three higher taxa and three guilds, respectively. The results of the post-hoc tests are presented in Suplementary Table S2.

In molluscs (Fig. 5a), we found significant differences in water content among the periods (*χ*^2^ = *8.73*, *p* = *0.013*); there was more water at the beginning than in the middle and at the end of overwintering.

In arachnids (Fig. 5b), lipid and glycogen content gradually decreased, while the water content gradually increased during overwintering, and values significantly differed by period (lipids: *χ*^2^ = *15.78*, *p* < *0.001*; glycogen: *χ*^2^ = *6.68*, *p* = *0.035*; water: *χ*^2^ = *18.06*, *p* < *0.001*). Lipid content was higher at the beginning than in the middle and at the end of overwintering, and glycogen content was higher at the beginning that at the end. Water content was higher in the middle and at the end than at the beginning of overwintering.

In insects (Fig. 5c), there were significant differences in lipid (*χ*^2^ = *14.70, p* = *0.001*) and water (*χ*^2^ = *7.73*, *p* = *0.021*) content during overwintering. Lipid content was higher at the beginning than in the middle and at the end of overwintering, while water content was higher at the end than in the middle of overwintering.

In Guild 1 (Fig. 6a), lipid (*χ*^2^ = *10.03*, *p* = *0.007*) and glycogen (*χ*^2^ = *7.14*, *p* = *0.028*) content differed significantly among overwintering periods. Lipid content was higher at the beginning of overwintering than in both following periods, and glycogen content at the beginning than at the end of overwintering.

In Guild 2 (Fig. 6b), there were significant differences in lipid (*χ*^2^ = *12.78*, *p* = *0.002*) and water (*χ*^2^ = *15.67*, *p* < *0.001*) content during overwintering. Lipid content was higher at the beginning and in the middle than at the end, and water content was higher in the middle and at the end than at the beginning of overwintering.

In Guild 3 (Fig. 6c), lipid (*χ*^2^ = *7.32*, *p* = *0.026*) and glycogen (*χ*^2^ = *6.28*, *p* = *0.043*) content differed significantly between periods. Lipid content was higher at the beginning than in the middle of overwintering, and glycogen content was higher in the middle and at the end than at the beginning of overwintering.

### Total amount of energy-supplying compounds and water content in higher taxa and guilds

**Figure 7:**
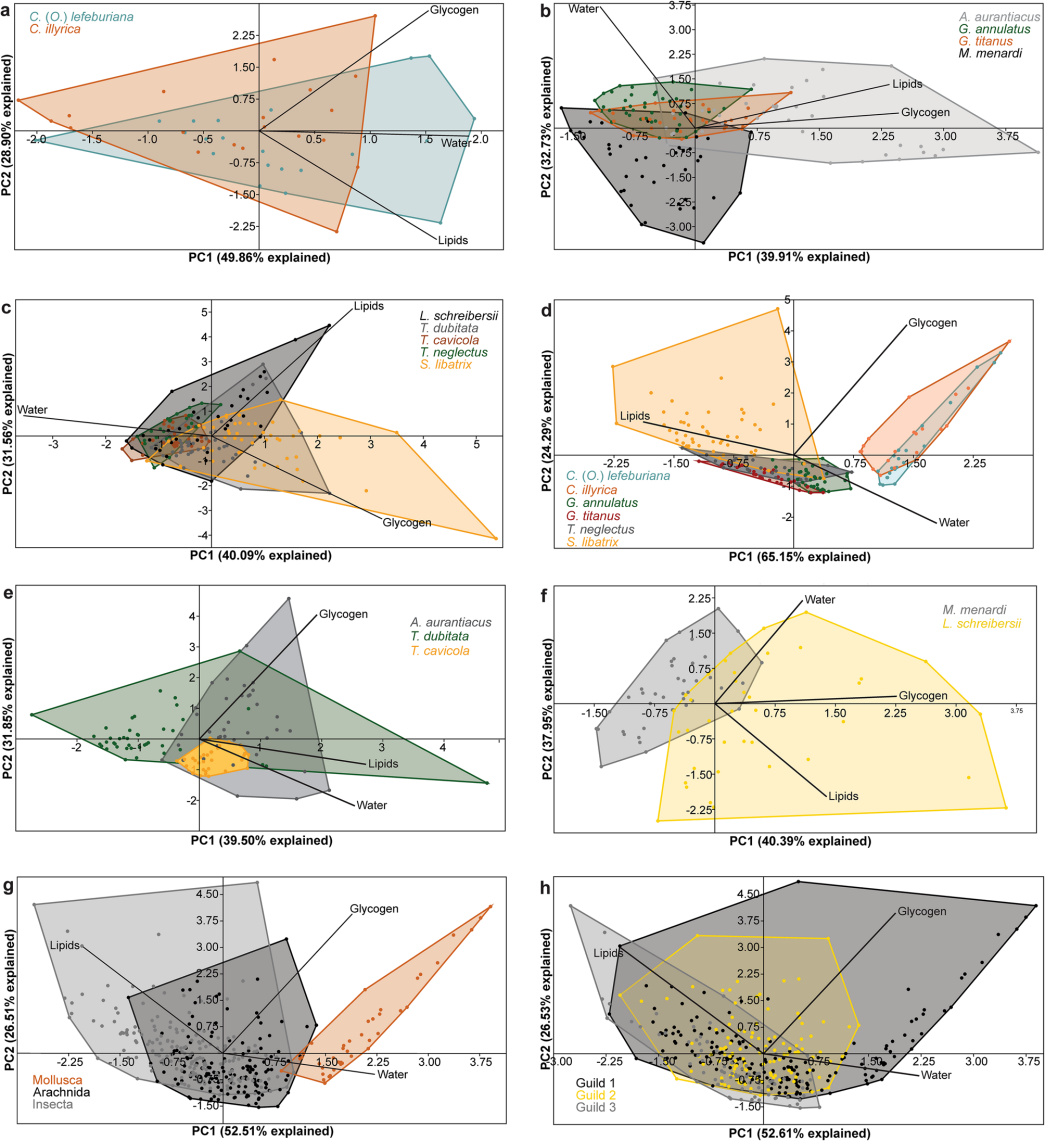
Bivariate plots with polygons for individual species of Mollusca (**a**), Arachnida (**b**), Insecta (**c**), Guild 1 (**d**), Guild 2 (**e**) and Guild 3 (**f**), and the summary plots of higher taxa (g) and guilds (h) including the corresponding species onto the first two PCs derived from ordination of lipid, glycogen, and water content. The percentage of explained variance by both PCs is given in parentheses. For guild characteristics see the subsection Species studied in the Methods.

Ordination diagrams in Fig. 7, based on PCA, provide general information on lipid, glycogen and water content for species, higher taxa and guilds during overwintering. The results of the post-hoc tests are presented in Tables 3 and 4.

**Table 3:** Pairwise post-hoc comparisons for differences in lipid, glycogen and water content among species within higher taxa, and among higher taxa; statistically significant differences are in bold.

	Lipids (Arachnida)			
	*A. aurantiacus*	*G. annulatus*	*G. titanus*	
*M. menardi*	***U* = *335.50*,* p* < *0.001***	*U* = *701.00*,* p* = *0.891*	***U* = *210.50*,* p* < *0.001***	
*A. aurantiacus*		***U* = *358.00*,* p* < *0.001***	*U* = *599.00*,* p* = *1.000*	
*G. annulatus*			***U* = *196.00*,* p* < *0.001***	
Glycogen (Arachnida)				
*M. menardi*	***U* = *63.00*,* p* < *0.001***	***U* = *113.00*,* p* < *0.001***	*U* = *523.00*,* p* = *1.000*	
*A. aurantiacus*		***U* = *235.00*,* p* < *0.001***	***U* = *43.00*,* p* < *0.001***	
*G. annulatus*			***U* = *90.00*,* p* < *0.001***	
Water (Arachnida)				
*M. menardi*	***U* = *492.00*,* p* = *0.005***	***U* = *99.00*,* p* < *0.001***	***U* = *226.00*,* p* < *0.001***	
*A. aurantiacus*		***U* = *352.00*,* p* < *0.001***	*U* = *594.00*,* p* = *1.000*	
*G. annulatus*			***U* = *171.00*,* p* < *0.001***	

	*T. neglectus*	*L. schreibersii*	*T. dubitata*	*S. libatrix*
Lipids (Insecta)				
*T. cavicola*	*U* = *246.00*,* p* = *0.332*	*U* = *448.00*,* p* = *0.901*	***U* = *395.00*,* p* = *0.044***	*U* = *474.00*,* p* = *0.068*
*T. neglectus*		*U* = *411.00*,* p* = *1.000*	***U* = *244.00*,* p* = *0.005***	*U* = *549.00*,* p* = *1.000*
*L. schreibersii*			***U* = *471.00*,* p* = *0.017***	*U* = *854.00*,* p* = *1.000*
*T. dubitata*				***U* = *482.00*,* p* < *0.001***
Glycogen (Insecta)				
*T. cavicola*	*U* = *272.00*,* p* = *0.913*	*U* = *386.00*,* p* = *0.146*	*U* = *521.00*, *p* = *1.000*	*U* = *698.00*, *p* = *1.000*
*T. neglectus*		*U* = *384.00*,* p* = *1.00*	*U* = *495.00*, *p* = *1.000*	*U* = *419.00*,* p* = *0.616*
*L. schreibersii*			*U* = *669.00*, *p* = *1.000*	***U* = *570.00*,* p* = *0.030***
*T. dubitata*				*U* = *776.00*,* p* = *0.612*
Water (Insecta)				
*T. cavicola*	*U* = *336.00*,* p* = *1.000*	***U* = *190.00*,* p* < *0.001***	***U* = *31.00*,* p* < *0.001***	***U* = *8.00*,* p* < *0.001***
*T. neglectus*		***U* = *130.00*,* p* < *0.001***	***U* = *30.00*,* p* < *0.001***	***U* = *4.00*,* p* < *0.001***
*L. schreibersii*			***U* = *448.00*,* p* = *0.008***	***U* = *213.00*,* p* < *0.001***
*T. dubitata*				***U* = *388.00*,* p* < *0.001***

	Lipids (Higher taxa)		Glycogen (Higher taxa)	
	Mollusca	Insecta	Mollusca	Insecta
Arachnida	***U* = *237.00*,* p* < *0.001***	***U* = *9345.50*,* p* < *0.001***	***U* = *349.00*,* p* < *0.001***	***U* = *11,802.00*,* p* = *0.040***
Mollusca		***U* = *48.00*,* p* < *0.001***		***U* = *256.00*,* p* < *0.001***

	Water (Higher taxa)			
	Mollusca	Insecta		
Arachnida	***U* = *461.00*,* p* < *0.001***	***U* = *7366.00*,* p* < *0.001***		
Mollusca		***U* = *244.00*,* p* < *0.001***

**Table 4:** Pairwise post-hoc comparisons for differences in lipid, glycogen and water content among species within guilds, and among guilds; statistically significant differences are in bold italics.

	*C. illyrica*	*G. annulatus*	*G. titanus*	*T. neglectus*	*S. libatrix*
Lipids (Guild 1)					
*C. (O.) lefeburiana*	*U* = *158.00*,* p* = *1.000*	***U = 19.00, p < 0.001***	***U = 00.00, p < 0.001***	***U = 1.00, p < 0.001***	***U = 22.00, p < 0.001***
*C. illyrica*		***U = 12.00, p < 0.001***	***U = 00.00, p < 0.001***	***U = 00.00, p < 0.001***	***U = 9.00, p < 0.001***
*G. annulatus*			***U = 196.00, p < 0.001***	***U = 113.00, p < 0.001***	***U = 226.00, p < 0.001***
*G. titanus*				*U* = *282.00*,* p* = *1.000*	*U* = *540.00*,* p* = *0.979*
*T. neglectus*					*U* = *549.00*,* p* = *1.000*
Glycogen (Guild 1)					
*C. (O.) lefeburiana*	*U* = *154.00*,* p* = *1.000*	***U = 2.00, p < 0.001***	***U = 00.00, p < 0.001***	***U = 4.00, p < 0.001***	***U = 104.00, p = 0.003***
*C. illyrica*		***U = 00.00, p < 0.001***	***U = 00.00, p < 0.001***	***U = 00.00, p < 0.001***	***U = 62.00, p < 0.001***
*G. annulatus*			***U = 90.00, p < 0.001***	*U* = *392.00*, *p* = *1.000*	*U* = *832.00*, *p* = *1.000*
*G. titanus*				***U = 85.00, p < 0.001***	***U = 179.00, p < 0.001***
*T. neglectus*					*U* = *419.00*, *p* = *0.923*
Water (Guild 1)					
*C. (O.) lefeburiana*	*U* = *154.00*, *p* = *0.933*	***U* = *00.00*,* p* < *0.001***	***U* = *00.00*,* p* < *0.001***	***U* = *00.00*,* p < 0.001***	***U* = *00.00*,* p* < *0.001***
*C. illyrica*		***U* = *175.00*,* p* = *0.006***	***U* = *63.00*,* p* = *0.042***	***U* = *74.00*,* p = 0.001***	***U* = *00.00*,* p* < *0.001***
*G. annulatus*			***U* = *171.00*,* p* = *0.006***	***U* = *233.00*,* p* = *0.009***	***U* = *10.00*,* p* < *0.001***
*G. titanus*				*U* = *266.00*,* p* = *1.000*	***U* = *9.00*,* p* < *0.001***
*T. neglectus*					***U* = *4.00*,* p* < *0.001***

	Lipids (Guild 2)		Glycogen (Guild 2)		
	*T. dubitata*	*T. cavicola*	*T. dubitata*	*T. cavicola*	
*A. aurantiacus*	***U* = *550.50*,* p* = *0.022***	*U* = *547.00*,* p* = *1.000*	***U* = *305.00*,* p* < *0.001***	***U* = *200.00*,* p* < *0.001***	
*T. dubitata*		***U* = *395.00*,* p* = *0.013***		*U* = *521.00*, *p* = *0.445*	

	Water (Guild 2)				
	*T. dubitata*	*T. cavicola*			
*A. aurantiacus*	***U* = *216.00*,* p* < *0.001***	*U* = *554.00*,* p* = *1.000*			
*T. dubitata*		***U* = *31.00*,* p* < *0.001***			

	Glycogen (Guilds)		Water (Guilds)		
	Guild 2	Guild 3	Guild 2	Guild 3	
Guild 1	*U* = *9713.00*,* p* = *1.000*	***U* = *3207.00*,* p* < *0.001***	***U* = *7768.00*,* p* = *0.001***	***U* = *4377.00*,* p* < *0.001***	
Guild 2		***U* = *1618.00*,* p* < *0.001***		*U* = *3804.00*,* p* = *0.136*	

In the two mollusc species, there was considerable overlap in lipid, glycogen, and water content levels during overwintering, and consequently, there were no statistically significant differences (Fig. 7a).

Among four arachnid species, significant differences were found in lipid (*χ*^2^ = *44.65*, *p* < *0.001*), glycogen (*χ*^2^ = *101.00*, *p* < *0.001*) and water (*χ*^2^ = *62.62*, *p* < *0.001*) content (Fig. 7b). *Amilenus auranticus* and *G. titanus* had significantly higher lipid content than *M. menardi* and *G. annulatus*. Considering glycogen content, *A. aurantiacus* and *G. annulatus* had significantly higher values than *M. menardi*, *A. aurantiacus* higher than *G. annulatus* and *G. titanus*, and *G. annulatus* higher than *G. titanus*. Water content in *A. aurantiacus*, *G. annulatus* and *G. titanus* was significantly higher than in *M. menardi*. In addition, *G. annulatus* had significantly higher water content than *G. titanus* and *A. aurantiacus*. Comparing the troglophilic *M. menardi* with the other three arachnids, we found that the lipid (*U* = *1247.00, p* < *0.001*), glycogen (*U* = *699.00*, *p* < *0.001*) and water content (*U* = *817.00*, *p* < *0.001*) were significantly lower in *M. menardi* than in these arachnids.

In insects, we also observed significant differences in lipid (*χ*^2^ = *25.68*, *p* < *0.001*), glycogen (*χ*^2^ = *12.38*, *p* = *0.015*) and water (*χ*^2^ = *121.40*, *p* < *0.001*) content (Fig. 7c). In *Troglophilus cavicola*, *T. neglectus*, *L. schreibersii* and *S. libatrix* lipid contents were significantly higher than in *T. dubitata*. Glycogen content in *S. libatrix* was significantly higher than in *L. schreibersii*. Water content in *T. cavicola* was significantly higher than in *L. schreibersii*, *T. dubitata* and *S. libatrix*. Moreover, water content in *T. neglectus* was significantly higher than in *L. schreibersii*, *T. dubitata* and *S. libatrix*. It was also higher in *L. schreibersii* than in *T. dubitata* and *S. libatrix*, and higher in *T. dubitata* than in *S. libatrix*. In testing for differences between troglophilic *L. schreibersii* and the other insect species under study, it was found that lipid content was significantly higher (*U* = *2184.00*, *p* = *0.049*), while glycogen content was significantly lower in *L. schreibersii* (*U* = *2009.00*, *p* = *0.010*) than in the other insects.

In Guild 1, there were significant differences in lipid (*U* = *121.00, p* < *0.001*), glycogen (*U* = *114.40, p* < *0.001*) and water (*U* = *148.20, p* < *0.001*) content among species (Fig. 7d). Lipid and glycogen contents were higher in *G. annulatus, G. titanus*, *T. neglectus* and *S. libatrix* than in *C.* (*O.*) *lefeburiana* and *C. illyrica*. In addition, lipid content was also higher in *G. titanus*, *T. neglectus* and *S. libatrix* than in *G. annulatus.* We found higher glycogen content in *G. titanus* than in *G. annulatus*, and in *T. neglectus* and *S. libatrix* than in *G. titanus*. Water content in *C.* (*O.*) *lefeburiana* and *C. illyrica* was higher than in the other species of this guild. It was also higher in *G. annulatus* than in *G. titanus*, *T. neglectus* and *S. libatrix*, and higher in *G. titanus* and *T. neglectus* than in *S. libatrix*.

In Guild 2, lipid (*χ*^2^ = *10.67, p* = *0.005*), glycogen (*χ*^2^ = *33.57, p* < *0.001*) and water (*χ*^2^ = *55.72, p* < *0.001*) contents differed significantly among species (Fig. 7e). Lipid and water content were higher in *A. aurantiacus* and *T. dubitata* than in *T. cavicola,* and glycogen content was higher in *A. aurantiacus* than in the other two species.

Among the two species in Guild 3, significant differences were found in lipid (*U* = *295.00, p* < *0.001*) and glycogen (*U* = *432.00, p* < *0.001*) content; both were higher in *L. scheibersii* than in *M. menardi* (Fig. 7f).

Among Mollusca, Arachnida and Insecta, we also found significant differences in lipid (*χ*^2^ = *123.40*, *p* < *0.001*), glycogen (*χ*^2^ = *95.53*, *p* < *0.001*) and water (*χ*^2^ = *134.50*, *p* < *0.001*) content (Fig. 7g). In insects, lipid content was significantly higher than in mollusks and arachnids, and in arachnids, lipid content was higher than in molluscs. On the other hand, in molluscs, glycogen content was higher than in arachnids and insects. Moreover, in insects, glycogen content was higher than in arachnids. In molluscs, water content was higher than in arachnids and insects. In contrast to glycogen content, in arachnids, water content was higher than in insects.

Among the three guilds, significant differences were observed in glycogen (*χ*^2^ = *68.81*, *p* < *0.001*) and water (*χ*^2^ = *31.94*, *p* < *0.001*) content (Fig. 7e). In species assigned to Guild 1 and Guild 2, glycogen content was higher than those of Guild 3. In addition, in species of Guild 1, water content was higher than in the species of the other two guilds.

### Transmission electron microscopy

**Figure 8:**
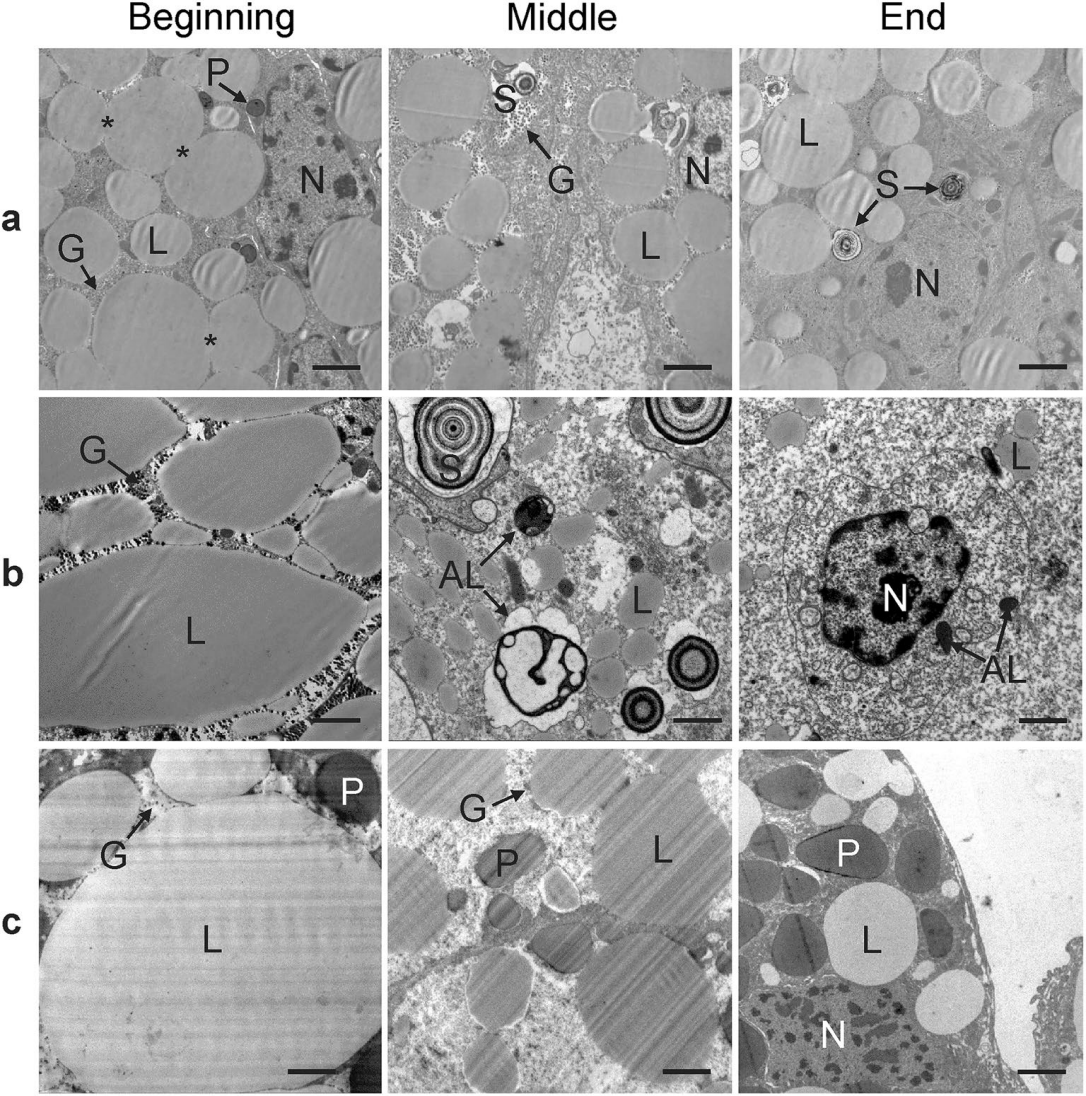
Selected illustrative cases of arthropods applying different overwintering strategies to show similarities and differences in intracellular changes during overwintering. Ultrathin section of the (**a**) midgut diverticulum of *A. aurantiacus*, (**b**) midgut diverticulum of *M. menardi*, and (**c**) fat body of *S. libatrix*. AL, autolysosome; G, glycogen; L, lipid droplet; N, nucleus; P, protein granulum; S, spherite. Asterisk, close contact of lipid droplets. Bars in all figures: 2 µm.

Figure 8 illustrates intracellular conditions in selected arachnids and insects. Presentation of gastropods is missing because of damaged slices, useless for the TEM analysis (see explanation in Methods).

In most species, the amount of reserve compounds diminished during winter. However, TEM reveals variation in the pattern of diminishing. In the harvestman *A. aurantiacus* (Fig. 8a), at the beginning of overwintering, the cytoplasm of adipocytes in the epithelial cells of the midgut diverticula was crowded with lipid droplets and glycogen granules. Consequently, the lipid droplets were in close contact, and many neighbouring droplets fused. Between the droplets, the cytoplasm was locally electron-dense because of numerous glycogen granules and protein granules. In the middle and at the end of overwintering, the diameter of lipid droplets decreased slightly.

In *M. menardi* (Fig. 8b), at the beginning of overwintering, the cytoplasm of adipocytes in the epithelium of the midgut diverticula was filled with large to huge lipid droplets and numerous glycogen granules. In the middle and at the end of overwintering, the size and abundance of both energy-storing compounds was reduced, and autophagic structures appeared.

In *S. libatrix* (Fig. 8c), at the beginning of overwintering, the adipocytes in the fat body were characterized by numerous huge lipid droplets, protein granules and glycogen granules. In the middle and at the end of overwintering, the size of the lipid droplets and protein granules and the abundance of the glycogen granules diminished.

## Discussion

In temperate zone in winter, many climatic conditions in caves, such as temperature and air humidity, are more favourable as compared to conditions on the surface. Consequently, many epigean animals take advantage of such conditions to find hibernacula in caves, all showing relatively high level of starvation hardiness in dormancy. As originally predicted, the responses of nine inactive and two active terrestrial species included in this study showed large differences in stores of energy-supplying compound and energy consumption during overwintering starvation. In addition, overwintering strategies were even more heterogeneous than predicted. Here we discuss the main findings.

### Dynamics of energy-supplying compounds and water content in the studied species during overwintering

Considering the fact that the eleven species studied belong to different higher taxa, we confirmed conspicuous differences in the response to starvation and in the consumption of reserve energy-supplying compounds during overwintering. The consumption of these compounds is strongly higher taxa-dependant; in the gastropods *C.* (*O.*) *lefeburiana* and *C. illyrica*, the energy-supplying stores consist almost exclusively of glycogen ^64,65^, in insects lipids strongly prevail, and in arachnids the use of both types of reserve compounds seems to be balanced.

Enhanced glycogen metabolism in both invertebrates and in vertebrates allows faster and more vigorous reaction to adverse stimuli than lipid metabolism does^49,66^. *Gyas annulatus*, e.g., which overwinter closely above a snow-water stream, accumulates more glycogen that the congeneric *G. titanus*, which overwinter in the same cave in unthreatened microhabitats; glycogen metabolism enables individuals of *G. annulatus* to flee quickly away from sudden water level rise^49^. Cave-dwelling individuals of the Mexican cavefish *Astyanax mexicanus* with enhanced glycogen metabolism show the same strategy in energy metabolism: they swim a few-times slower, but reach maximum swimming speed a few times faster than their predominantly lipid-fuelled riverine counterparts^66^. Thus, enhanced glycogen metabolism might have an adaptive role for quick reaction to threating events in subterranean habitats.

Arachnids and insects can store a great amount of lipid reserves in the form of cytoplasmic lipid droplets in adipocytes^16,24^. Lipids are the most economical supplies for long-term starvation, and are therefore primarily consumed in overwintering and exclusively subterranean residents^17,24^. The highest consumption in *S. libatrix* and *T. neglectus* indicates that metabolic depression^67^ is limited.

The apparently surprising accumulation of energy-supplying compounds in *L. schreibersii* during winter perfectly illustrates the opportunistic feeding habit of species adapted to conditions in caves, generally considered extreme habitat^11,34^, which mostly refers to a limited and erratic food supply. *Laemostenus schreibersii* feed continuously during first half of winter, e.g., on guanobionts, until the adverse conditions dictate cessation. Thus, this beetle optimally exploit food when available. Then, they probably move from the cave into the adjacent fissure network and remain active^68^, or, alternatively, they enter torpor—a postponed dormancy—as they do in laboratory below 6–7 °C (own unpublished data), which might correspond to quiescence.

Because of glycolysis, water content decreases during winter in glycogen-fuelled dormant organisms, which is well evident in our two gastropod species from the beginning to the end of overwintering. The increase in water content in *C.* (*O.*) *lefeburiana* must be considered an artefact, since these snails apparently consumed water before being sampled at the end of overwintering. In contrast, lipolysis produces metabolic water, which contributes to a higher water content^55^ as found in our dormant arachnids and insects. A net loss of body water in glycogen-fuelled organisms, and its net gain in lipid-fuelled organisms well comply with the findings on the consuption of energy-supplying reserves and vice versa. Thus, trends in water content are of supplementary importance for the interpretation of the main energy-fuelling processes in these organisms. All these findings must be taken into account when considering species of different higher taxa.

### Energy consumption in the studied species during overwintering

Species in diapause (Guild 1), i.e., *C.* (*O.*) *lefeburiana*, *C. illyrica*, *G. annulatus*, *G. titanus*, *S. libatrix* and *T. neglectus*, that do not move and develop during overwintering, were consequently expected to have low consumption of energy. However, this was not the case for.

*T. neglectus*^25^ and *S. libatrix*^20^. The high energy consumption is most likely due to poorly suppressed basal metabolic rate during overwintering, which could indicate poor energetic adaptation to the habitat. All other species in the guild show very cautious spending of energy for the basal metabolic rate.

On the other hand, species with ongoing ontogenesis under fasting conditions (Guild 2), i.e., *A. aurantiacus*^44,54^, *T. dubitata*^53^ and *T. cavicola*^55,56^, were accordingly expected to have high energy consumption. *Amilenus aurantiacus* and *T. dubitata* show low energy consumption, although maturating during overwintering. This might indicate a better energetic adaptation of these species to the habitat. However, high energy consumption in *T. cavicola* was expected because of maturation process.

Moderate consumption was expected for the two, year-round active, opportunistic feeding species (Guild 3), i.e., *M. menardi* and *L. schreibersii*. *Meta menardi* showed a relatively low energy consumption, because they occasionally feed in winter^45,50,51^ and in this way replenish the energy reserves that were consumed while food was not available. Thus, *M. menardi* undergo a compulsory overwintering starvation at times of scarce opportunity to feed^56^, which might partly correspond to hypometabolism as a mode of dormancy^67^. In accordance with extending feeding in winter, *L. schreibersii* accumulated about 1 J/g energy per day.

Lipids were found to be the major energy-supplying compounds^24^, whereas glycogen metabolism should be studied in more detail, as the utilization of triacylglycerides may be associated with glycogen resynthesis^40^ for either glucose release or moulding processes^18^. Our results suggest that energetic strategies evolve independently; each overwintering species deserves separate consideration with this respect. Accordingly, we did not confirm our hypothesis on co-evolution of functional and energetic traits.

### Cytological findings

A cytological approach allowed visual comparison of the size and relative abundance of energy-supplying compounds in selected tissues or organs. Similar changes were observed in all three species assessed by TEM, which we attribute to the consumption of energy-supplying compounds during overwintering. Lipid droplets in the cells decreased in size and abundance, and glycogen and protein granules decreased in abundance; any variation in the glycogen granules cannot be assessed by TEM micrographs. In addition, autophagic structures appeared in *M. menardi* in the middle and at the end of overwintering, providing an additional source of energy in this and some other overwintering species, e.g.^44,45^. The cytological evidence of reserve compounds was in complete agreement with the biochemical findings, and was, thus, of supporting importance.

### Artefact outcomes

The sampling approach used in this study may lead to some artifactual results, which could be particularly evident when considering individual species on a fine scale. Inherent variability within the species may be a consequence of the specific life history of the individuals collected, such as differences in feeding opportunities, stage and timing of reproduction, food availability and microclimatic conditions in overwintering sites. Some other data suggest inappropriate timing of sampling, as in the case of *C.* (*O.*) *lefeburiana*, which obviously began to feed before sampling, given the high water content at the end of overwintering. On the other hand, in many species, e.g., in *T. cavicola*, the increase in glycogen rate alone at the end of overwintering indicates changes in metabolic activity before entering anew epigean, active ecophase in the life cycle. Despite such cases, the general trends considered here remain well established, as confirmed in experimentally designed studies^21,50^. Differences in the measuring methods of lipid and glycogen amounts, e.g.^25,69–71^, negligibly affect the large-scale comparative results.

## Concluding remarks

With the exception of *L. schreibersii*, which abundantly feed in caves in winter, nine other studied non-troglobiont species undergo a programmed periodical starvation, and *M. menardi* a compulsory starvation. However, starvation hardiness is the only collective trait in these ten species, an obligate, preadaptive trait for life in the subterranean environments. This preadaptation might evolve into a permanent adaptation, as experienced in troglobionts, by economizing the use of energy-supplying compounds and energy. Another two important steps in adaptation to permanent life in the habitat are the start of feeding and completion of the reproduction in the habitat. The first applies to *M. menardi*, and the second might apply to *L. schreibersii*. While studies on *M. menardi* well compile, very little is known about the biology and ecology of *L. schreibersii* and many other species studied. It is a great challenge to fulfill these knowledge gaps in the future in these and in other non-troglobiont species, e.g.^72–75^.

## Supplementary Information

Supplementary Tables.

## Data Availability

The datasets generated during and/or analysed during the current study are available from the corresponding author on reasonable request.
