# Supplementary material for: Starvation hardiness as preadaptation for life in subterranean habitats
Source: Sci Rep. 2023 Jun 14;13:9643. doi: 10.1038/s41598-023-36556-9 (PMC10267165; doi:10.1038/s41598-023-36556-9)
Supplement: Supplementary file 1 — Supplementary Tables. [file 41598_2023_36556_MOESM1_ESM.docx]

**Supplementary Table S1.** Pairwise post-hoc comparisons for differences in lipid, glycogen and water content among the three overwintering periods for individual species; statistically significant differences are in bold.

| **Species** | **Variable** | **Beginning vs. Middle** | **Middle vs. End** | **End vs. Beginning** |
| --- | --- | --- | --- | --- |
| *C. (O.) lefeburiana* | Glycogen | *Q=0.18*, *p=0.992* | ***Q=4.98*, *p=0.007*** | ***Q=6.02*, *p=0.001*** |
|  | Water | *Q=1.25*, *p=0.656* | ***Q=6.46*, *p=0.001*** | ***Q=4.37*, *p=0.016*** |
| *C. illyrica* | Lipids | *Q=2.55*, *p=0.199* | ***Q=3.62*, *p=0.049*** | *Q=0.93*, *p=0.791* |
|  | Water | ***U=1.00*, *p=0.012*** | ***U=0.00*, *p=0.008*** | ***U=0.00*, *p=0.037*** |
| *A. aurantiacus* | Lipids | *Q=0.78*, *p=0.848* | ***Q=4.66* , *p=0.006*** | ***Q=4.71* , *p=0.006*** |
|  | Glycogen | ***U=7.00*, *p<0.001*** | ***U=34.00*, *p=0.012*** | *U=26.00*, *p=0.227* |
|  | Water | ***Q=15.89*, *p<0.001*** | *Q=0.75*, *p=0.858* | ***Q=14.40*, *p<0.001*** |
| *G. annulatus* | Glycogen | ***Q=7.47*, *p=0.017*** | ***Q=7.50*, *p=0.016*** | ***Q=12.96*, *p<0.001*** |
| *G. titanus* | Glycogen | *Q=2.53*, *p=0.193* | ***Q=5.37*, *p=0.002*** | ***Q=7.89*, *p=0.019*** |
|  | Water | *Q=2.95*, *p=0.111* | *Q=3.18*, *p=0.081* | ***Q=6.14*, *p=0.001*** |
| *M. menardi* | Lipids | ***U=24.00*, *p=0.003*** | *U=86.00*, *p=0.418* | *U=33.00*, *p=0.242* |
| *L. schreibersii* | Glycogen | *Q=3.04*, *p=0.095* | ***Q=4.45*, *p=0.009*** | ***Q=6.46*, *p<0.001*** |
| *T. dubitata* | Glycogen | *U=68.00*, *p=0.369* | ***U=29.00*, *p=0.002*** | ***U=0.00*, *p<0.001*** |
| *S. libatrix* | Lipids | ***U=11.00*, *p<0.001*** | *U=127.00*, *p=1.000* | ***U=13.00*, *p=0.017*** |
| *T. cavicola* | Lipids | *Q=0.60*, *p=0.906* | ***Q=4.12*, *p=0.018*** | ***Q=3.62*, *p=0.042*** |
|  | Water | ***Q=3.53*, *p=0.048*** | ***Q=12.44*, *p<0.001*** | ***Q=16.26*, *p<0.001*** |
| *T. neglectus* | Lipids | ***Q=3.74*, *p=0.039*** | *Q=2.95*, *p=0.117* | ***Q=7.02*, *p<0.001*** |

**Supplementary Table S2.** Pairwise post-hoc comparisons for differences in lipid, glycogen and water content among the three overwintering periods for higher taxa and guilds; statistically significant differences are in bold.

|  | **Variable** | **Beginning vs. Middle** | **Middle vs. End** | **End vs. Beginning** |
| --- | --- | --- | --- | --- |
| **Higher taxa** | | | | |
| **Mollusca** | Water | ***U=33.00*, *p=0.003*** | *U=98.00*, *p=1.000* | ***U=25.00*, *p=0.038*** |
| **Arachnida** | Lipids | ***U=953.00*, *p=0.013*** | *U=1329.50*, *p=1.000* | ***U=393.50*, *p<0.001*** |
|  | Glycogen | *U=1129.00*, *p=0.093* | *U=1209.00*, *p=0.117* | ***U=591.00*, *p=0.021*** |
|  | Water | ***U=826.00*, *p=0.001*** | *U=1336.00*, *p=1.000* | ***U=417.00*, *p<0.001*** |
| **Insecta** | Lipids | ***U=1423.00*, *p=0.007*** | *U=1777.00*, *p=0.477* | ***U=712.00*, *p=0.002*** |
|  | Water | *U=1926.00*, *p=1.000* | ***U=1501.00*, *p=0.022*** | *U=925.00*, *p=0.152* |
| **Guilds** | | | | |
| **Guild 1** | Lipids | ***U=1517.00*, *p=0.027*** | *U=1850.00*, *p=0.852* | ***U=806.00*, *p=0.015*** |
|  | Glycogen | *U=1879.00*, *p=1.000* | *U=1629.00*, *p=0.109* | ***U=853.00*, *p=0.041*** |
| **Guild 2** | Lipids | *U=668.00*, *p=0.730* | ***U=533.00*, *p=0.042*** | ***U=220.50*, *p=0.001*** |
|  | Water | ***U=474.00*, *p=0.008*** | *U=600.00*, *p=0.208* | ***U=232.00*, *p=0.001*** |
| **Guild 3** | Lipids | ***U=211.00*, *p=0.019*** | *U=404.00*, *p=1.000* | *U=141.00*, *p=0.233* |
|  | Glycogen | ***U=246.00*, *p=0.030*** | *U=388.00*, *p=0.449* | ***U=126.00*, *p=0.031*** |
